# Advances in Bispecific Antibodies and Antibody–Drug Conjugates for Colorectal Cancer Treatment

**DOI:** 10.3390/antib15050080

**Published:** 2026-09-01

**Authors:** Maya G. Cappellino, Sean P. Sullivan, Peyton C. High, Tiffani A. Blackburn, Kendra S. Carmon

**Affiliations:** 1 Center for Translational Cancer Research, The Brown Foundation Institute of Molecular Medicine, University of Texas Health Science Center at Houston, Houston, TX 77030, USA; maya.cappellino@uth.tmc.edu (M.G.C.); sean.sullivan@uth.tmc.edu (S.P.S.); peyton.high@uth.tmc.edu (P.C.H.); tiffani.blackburn@uth.tmc.edu (T.A.B.); 2 Graduate School of Biomedical Sciences, The University of Texas MD Anderson Cancer Center and UTHealth Houston, Houston, TX 77030, USA

**Keywords:** antibody–drug conjugates, colorectal cancer, bispecific antibody, bispecific antibody–drug conjugates

## Abstract

Bispecific antibodies (bsAbs) and bispecific antibody–drug conjugates (bsADCs) represent promising classes of emerging targeted therapeutics with the potential to overcome tumor heterogeneity and resistance in colorectal cancer (CRC). BsAbs can simultaneously engage multiple tumor antigens or immune cells or bind two distinct epitopes within a single target, enabling mechanisms of action beyond the capabilities of monoclonal antibodies. Bispecific T cell engagers facilitate targeted destruction of tumors through immune cell recruitment, while dual immune checkpoint inhibitors enhance immune activation by blocking T cell inhibitory signals. Furthermore, bsAbs can mediate dual signaling pathway inhibition through binding multiple receptor tyrosine kinase receptors or other tumor cell surface proteins. BsADCs integrate the dual-antigen recognition of bsAbs with targeted payload delivery, utilizing receptor-mediated endocytosis to deliver potent cytotoxic payloads selectively to CRC cells, while minimizing systemic toxicity. Recent advances in bsAb engineering, linker chemistry, site-specific conjugation, and payload design have accelerated the development of bsADCs for solid tumors, including CRC. BsAbs and bsADCs provide opportunities to improve tumor selectivity, enhance internalization, overcome antigen escape, and expand the population of CRC patients eligible for targeted therapy. Emerging preclinical studies demonstrate encouraging anti-tumor activity for bispecific modalities in CRC, while early clinical trials are beginning to establish their translational potential. This review summarizes the current landscape of bsAbs and bsADCs in therapeutic development for CRC, highlighting key biological targets, engineering strategies, mechanisms of action, and clinical status. We also discuss the major challenges facing clinical translation and provide perspectives on future directions for bispecific therapies in CRC.

## 1. Introduction

Colorectal cancer (CRC) remains a major global health burden and is the second-leading cause of cancer-related mortality among men and women combined, ranking only behind lung cancer [1]. Sporadic-onset CRC commonly develops through sequential acquisition of mutations in key tumor suppressor genes (e.g., *APC*, *SMAD4*, and *TP53*) and oncogenes (e.g., *KRAS*, *PI3KCA*, and *BRAF*) [2]. In addition to genetic components, other factors including advanced age, dietary habits, obesity, sedentary lifestyle, alcohol consumption, smoking, and underlying inflammatory bowel diseases all influence individual predisposition to CRC [1]. Hereditary conditions including Lynch syndrome, associated with germline mutations in DNA mismatch repair genes (*MLH1*, *MSH2*, *MSH6*, and *PMS2*), and familial adenomatous polyposis, caused by mutations in the *APC* tumor suppressor gene, contribute to a smaller but clinically significant subset of cases [2]. Concerningly, while overall CRC incidence has decreased over the last 20 years, increasing prevalence among patients under 50 years of age highlights a shift in disease demographics and emphasizes the growing need for improved prevention and treatment strategies for CRC [1].

Current standard-of-care treatment for CRC is largely determined by disease stage and may include surgical resection, chemotherapy, radiotherapy, and/or monoclonal antibody (mAb)-based targeted therapies [3]. For localized disease, surgical tumor resection is often curative and typically followed by adjuvant chemotherapy such as FOLFOX (folinic acid, fluorouracil, and oxaliplatin) or FOLFIRI (folinic acid, fluorouracil, and irinotecan) to reduce risk of recurrence [3]. In cases of advanced or metastatic (mCRC), chemotherapy regimens are commonly combined with agents directed against epidermal growth factor receptor (EGFR; e.g., cetuximab, panitumumab) or vascular endothelial growth factor (VEGF; e.g., bevacizumab) depending on tumor sidedness and *RAS/BRAF* mutation status [4,5]. Immune checkpoint inhibitors have also demonstrated efficacy in a small subset of patients with mismatch repair-deficient (dMMR) or microsatellite instability-high (MSI-high) tumors [5,6] (~12–15% of all CRC and ~4–5% of mCRC). Despite these advances, the overall prognosis for mCRC remains poor, with a 5-year relative survival rate of only ~15% and frequent development of therapeutic resistance [1].

A major limitation of current treatment strategies is their inability to adequately address the significant molecular and cellular heterogeneity of CRC. CRC tumors often harbor diverse genetic mutations and dynamically interact with the tumor microenvironment, contributing to variable therapeutic responses and resistance mechanisms [7]. Furthermore, conventional chemotherapy is associated with significant systemic toxicity and lacks tumor specificity, limiting long-term utility and tolerability. These shortcomings have driven a shift toward precision medicine approaches that tailor therapies to tumor biology and exploit specific molecular vulnerabilities.

In part, cancer stem-like cells (CSCs), a small subpopulation of cells within a tumor defined by infinite replicative potential and the ability to initiate tumorigenesis, are a major driver of tumor progression, therapeutic resistance, and metastasis in CRC and other tumors [8,9]. Several cell surface markers have been established to define CSCs in CRC, including leucine-rich repeat-containing G protein-coupled receptor 5 (LGR5), prominin-1 (CD133), cluster of differentiation 44 (CD44), activated leukocyte cell adhesion molecule (ALCAM; CD166), and epithelial cell adhesion molecule (EpCAM), among others [10]. Mounting evidence indicates that CSCs exhibit highly dynamic cell state shifts between stem- and non-stem cell-like states in response to therapeutic pressure and microenvironmental stimuli, substantially contributing to tumor heterogeneity and therapeutic evasion. Given their critical roles in tumor progression and therapeutic response, CSC-targeting therapeutic approaches have been pursued to improve CRC treatment outcomes, resulting in the initiation of several clinical trials with therapeutic modalities including cancer vaccines, chimeric antigen receptor (CAR) T cells, and antibody-based approaches [9].

Antibody-based therapies have emerged as a promising strategy to improve selectivity and efficacy in several cancer types. While EGFR- and VEGF-targeting mAbs have demonstrated clinical benefit in CRC, they are often restricted to specific patient subgroups (e.g., *KRAS* wild-type tumors), and many patients either fail to respond or eventually develop resistance and relapse [11,12,13]. Though the CSC-targeting LGR5 mAb BNC101 (NCT02726334) entered clinical development, phase I trials were eventually terminated following completion of dose escalation for unreported reasons. Antibody–drug conjugates (ADCs) consist of mAbs linked to cytotoxic payloads and build upon the clinical success of mAb therapeutics by combining mAb-mediated tumor selectivity with payload-mediated cell death [14,15,16]. Although 15 ADCs have been approved clinically, trastuzumab deruxtecan (T-DXd) is the only ADC currently approved for CRC and its use is limited to human epidermal growth factor receptor 2 (HER2)-amplified mCRC (~4% of cases) [14,15,16]. CSC-targeting ADCs, such as those targeting LGR5 or EpCAM (NCT06265688) [17], have demonstrated promise in pre-clinical CRC models and phase I clinical trials, respectively, though the clinical exploration of CSC-targeting ADCs remains largely in its infancy.

Bispecific antibodies (bsAbs) and bispecific ADCs (bsADCs) build on the successes of mAbs and monospecific ADCs and represent a next-generation of therapeutics designed to simultaneously engage two distinct targets or antigen epitopes. These bispecific agents can redirect immune effector cells toward tumor cells or co-target complementary signaling pathways to enhance antitumor activity and overcome resistance associated with single-target therapies [18]. In addition to dual-antigen targeting, bsADCs deliver a potent cytotoxic payload directly to tumor cells following receptor-mediated internalization, providing an additional mechanism to eliminate cancer cells. Given the complexity and heterogeneity of CRC, dual-targeting strategies offer an encouraging avenue for improving therapeutic outcomes. Further, given that CSC markers often also define normal intestinal stem cells (ISCs), bispecific-based approaches may prove more useful in specifically targeting CSCs while sparing normal ISCs by co-targeting tumor-enriched antigens. In this review, we will provide an overview of the current clinical and preclinical landscape of bsAb and bsADC therapeutics in CRC, targeting both CSC and non-CSC markers, and discuss future directions and limitations associated with these approaches.

## 2. Bispecific Antibodies (bsAbs)

BsAbs are an emerging class of biologics designed to simultaneously bind two distinct antigens or epitopes, enabling sophisticated functional capabilities beyond those of traditional monospecific mAbs [18]. To date, 16 bsAbs have received regulatory approval worldwide, 15 of which are indicated for cancer [18,19]. BsAbs can integrate multiple mechanisms of action within a single molecular entity such as dual pathway inhibition, immune cell recruitment, or dual immune checkpoint inhibition. Certain bsAbs, including biparatopic antibodies that target multiple epitopes on the same antigen, can promote receptor clustering, internalization, and downregulation, thereby enhancing inhibition of oncogenic signaling [20]. Notably, bsAbs often exert obligate mechanisms of action that require simultaneous target engagement that are not achievable by a combination of two mAbs [21]. Additionally, by actively engaging complementary targets, bsAbs may overcome compensatory signaling pathway activation and antigen loss, which are common limitations of mAb-based treatments [22]. Taken together, the integration of two targets allows for novel therapeutic mechanisms of bsAbs that may enhance efficacy as compared to single-target approaches.

### 2.1. BsAb Structural Formats and Design Considerations

**Figure 1:**
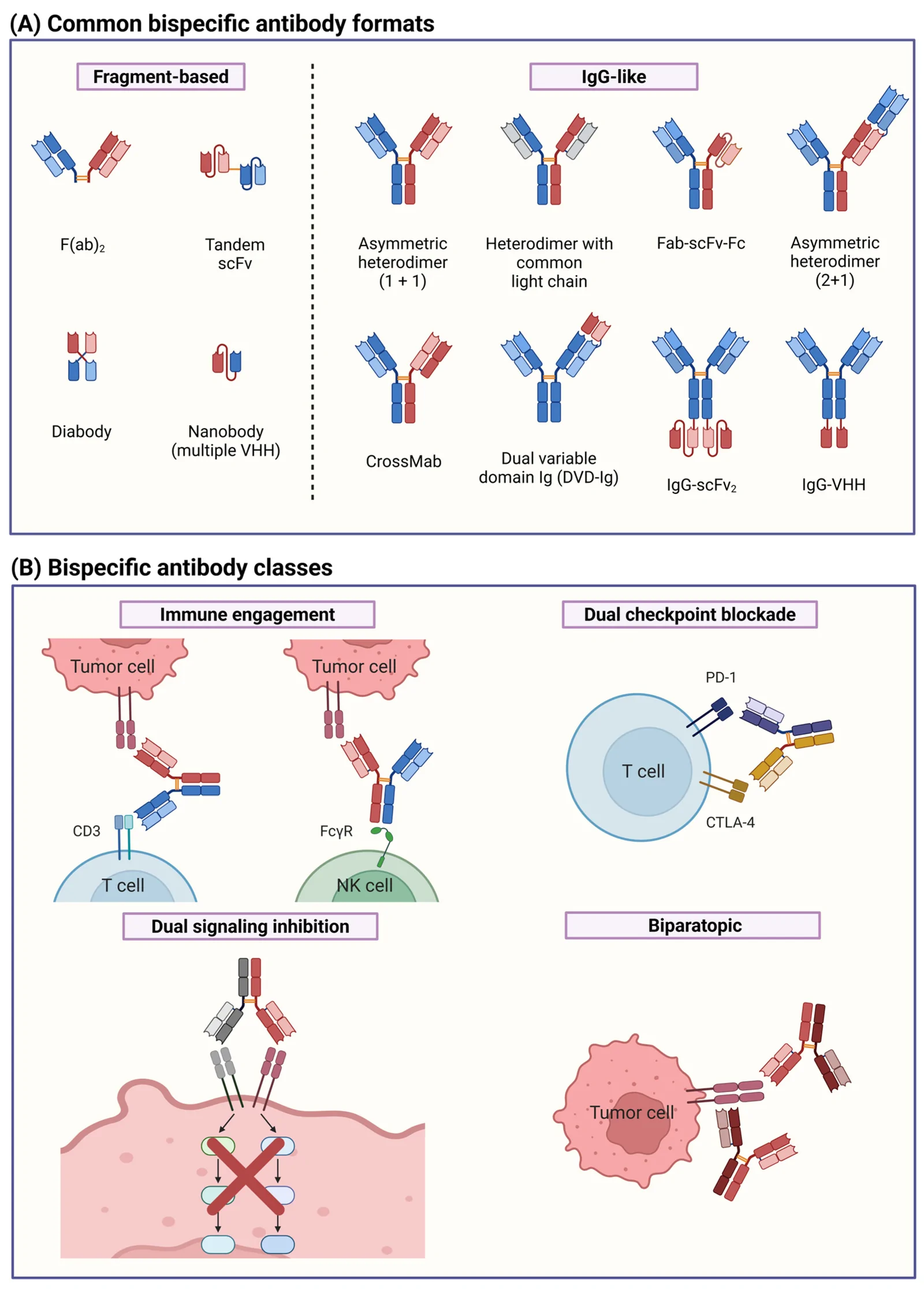
Common bispecific antibody formats, classes, and mechanisms of action. (**A**) The two general formats for bsAbs are fragment-based and IgG-like, though numerous variations in these formats can be generated. (**B**) BsAbs can also be categorized by class based on biological functions, including engagement of the immune system by T cell modulation or Fc interaction with FcyR on effector cells, dual checkpoint blockade, or dual signaling inhibition through targeting different tumor antigens. BsAbs may also enable biparatopic engagement, targeting multiple epitopes on the same antigen at the tumor cell surface.

BsAbs exhibit substantial structural diversity but can be broadly categorized into fragment-based formats lacking a fragment crystallizable (Fc) region and IgG-like designs that retain the Fc and its related effector functions (Figure 1), including antibody-dependent cellular cytotoxicity (ADCC), complement-dependent cytotoxicity (CDC), and antibody-dependent cellular phagocytosis (ADCP) [18,19]. Select examples of bsAbs are shown in Figure 1A, though over 100 different bsAb formats have been reported.

For IgG-like formats, Fc-driven immune mechanisms can potentially enhance antitumor activity by recruiting innate immune effector cells such as natural killer (NK) cells, macrophages, and complement components (Figure 1B). However, they may also introduce safety liabilities, such as those associated with immune activation and cytokine release syndrome, thereby necessitating careful engineering of Fc functionality to balance efficacy and tolerability [23]. For example, the IgG1 Fc may be mutated at various sites (e.g., L234, L235, N297) to reduce immune cell activation or, alternatively, engineered to enhance effector function through various mechanisms (e.g., reducing Fc fucosylation) [19]. Alternatively, different IgG classes, such as IgG4, which exhibits poor binding to Fc gamma receptor (FcγR) and C1q, may be employed in bsAb design to modulate immune cell activation [24]. Compared to larger IgG-like formats, fragment-based constructs, such as single-chain variable fragments (scFvs), offer advantages such as improved tissue penetration, increased design flexibility, and a potential solution to mispairing challenges during antibody assembly. IgG-like bsAbs typically provide improved stability, longer half-life, and compatibility with established manufacturing platforms [18,19]. The half-life of fragment-based bsAbs may be improved via the addition of an Fc or albumin-binding domain, highlighting the flexibility of bsAb design.

Bispecific modalities are commonly engineered through various mechanisms, including antibody domain fusions (e.g., scFv-Fab-Fc), point mutations to promote heavy chain (HC) heterodimerization and enforce correct light chain (LC) pairing, domain swapping, and modular assembly strategies that better control binding orientation and valency [19]. Example strategies for inducing proper HC pairing include DEKK and knobs-to-holes, while the CrossMab platform is utilized for LC pairing. The DEKK platform incorporates CH3 Fc-region amino acid substitutions that form stabilizing salt-bridge interactions to facilitate correct heterodimer formation and reduce mispairing of antibody chains during production [25]. The knobs-to-holes approach promotes HC heterodimerization by introducing complementary mutations within the CH3 domains by which a small amino acid is substituted for a larger one to create the “knob” and vice versa to generate the corresponding “hole” [26]. The CrossMab platform uses domain crossover engineering to promote correct LC pairing by exchanging selected domains in the Fab region [27]. Selection of bsAb format has important implications for pharmacokinetics, tolerability, and manufacturability [26,27]. More complex or asymmetric designs may introduce challenges such as reduced stability or increased immunogenicity due to non-native structural features. Conversely, simpler and more “IgG-like” formats are generally better tolerated and easier to produce at larger scales [19].

### 2.2. BsAb Mechanisms of Action

**Figure 2:**
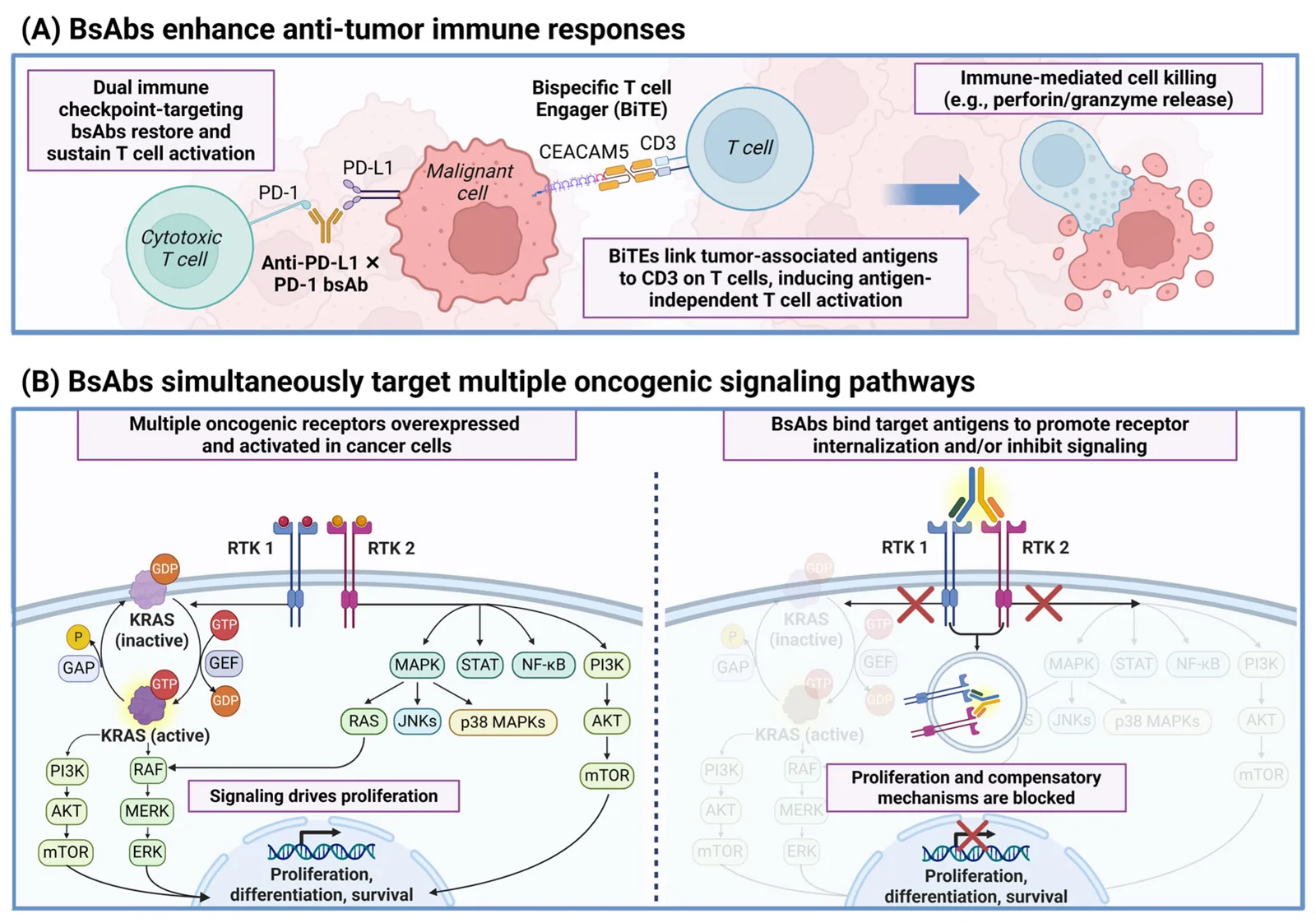
Bispecific antibodies overcome common CRC resistance mechanisms. (**A**) Immune-targeting bsAbs enhance anti-tumor responses by inducing and sustaining T cell activation. (**B**) BsAbs reduce the impact of target downregulation by modulating signaling at multiple nodes and may limit compensatory signaling by simultaneously targeting several tumor antigens.

The therapeutic activity of bsAbs is driven by several distinct and occasionally overlapping mechanisms. Many clinically approved bsAbs are bispecific T cell engagers (BiTEs), in which a bsAb simultaneously binds a tumor-associated antigen and a T cell-specific marker to facilitate immune synapse formation and targeted cytotoxicity independent of major histocompatibility complex (MHC)-mediated antigen presentation [28] (Figure 2A). Classical BiTEs are compact tandem scFv constructs lacking an Fc domain, consisting of one scFv directed against a tumor-associated antigen and the other against CD3 on T cells [29]. For example, carcinoembryonic antigen-related cell adhesion molecule 5 (CEACAM5/CEA) × CD3 BiTEs recruit and activate T cells through CD3 while simultaneously binding CEACAM5 on tumor cells, resulting in targeted cytotoxicity [28]. Dual immune checkpoint inhibitors represent another bsAb class and simultaneously target two immunoregulatory pathways to enhance antitumor immune responses and overcome mechanisms of immune suppression (Figure 2A). BsAb-mediated programmed cell death protein 1/programmed cell death ligand (PD-1/PD-L1) blockade has been shown to prevent tumor-mediated T cell exhaustion [30]. BsAbs integrating cytotoxic T-lymphocyte-associated protein 4 (CTLA-4) inhibition is another common approach, promoting early T cell priming and clonal expansion with the goal of generating more robust and durable immune responses than monotherapy approaches [31]. By simultaneously modulating complementary immune pathways, these bsAbs may also reduce reliance on pre-existing antitumor immunity and could improve responses in immunologically “cold” tumors such as microsatellite-stable (MSS) CRC.

In addition to immune modulation, bsAbs can directly alter tumor cell signaling and/or target CSCs through simultaneous engagement of multiple receptors to mediate concurrent blockade of two oncogenic signaling pathways. Although this strategy has been most extensively explored with receptor tyrosine kinases (RTKs) such as c-MET, HER2, and human epidermal growth factor receptor 3 (HER3), bsAbs can also target other classes of cell-surface proteins, including G protein-coupled receptors (GPCRs), cell adhesion molecules, and immune regulatory proteins that frequently contribute to acquired resistance following monospecific therapies. Additionally, bsAbs can promote enhanced receptor clustering, internalization, and downregulation. For example, HER2 biparatopic antibodies and EGFR × c-MET bsAb amivantamab have been shown to induce receptor clustering and greater internalization than conventional mAbs, contributing to more effective receptor downregulation and signaling inhibition [20,32]. By limiting alternative pathway activation, these dual-targeting strategies have the potential to address key mechanisms of therapeutic resistance in CRC (Figure 2B).

From a trafficking and cellular processing perspective, bsAbs targeting surface receptors often undergo receptor-mediated internalization, leading to receptor degradation or recycling. BsAbs may function by “and”- or “or”-gated mechanisms wherein internalization requires simultaneous binding to both receptors or either receptor alone, respectively [18]. Rapidly internalizing receptors may also be leveraged to facilitate internalization and subsequent degradation of otherwise surface-resident proteins or those that more slowly internalize [33,34]. Collectively, these mechanisms highlight the versatility of bsAbs as multifunctional and adaptable therapeutic platforms capable of addressing the complex tumor biology associated with CRC.

The wide array of bsAb mechanisms of action highlights the importance of target selection for bsAb design. Target selection requires careful consideration of antigen abundance, co-expression patterns, internalization properties, and tumor specificity. Importantly, the intended therapeutic mechanism guides target choice: signaling pathway-blocking bsAbs require complementary signaling nodes, whereas immune-engaging bsAbs require tumor-associated antigen and immune effector cell pairing.

## 3. Bispecific Antibody–Drug Conjugates (bsADCs)

BsADCs represent an integration of both bsAb and ADC technologies by combining dual-targeting specificity with the targeted delivery of potent cytotoxic payloads [14]. Similar to traditional ADCs, bsADCs consist of antibodies linked to cytotoxic payloads via cleavable or non-cleavable linkers, but they uniquely incorporate two or more antigen-binding domains to enhance tumor selectivity and improve targeting of tumor heterogeneity [35,36]. This dual specificity allows bsADCs to preferentially bind tumor cells overexpressing one or both target antigens, potentially reducing on-target, off-tumor toxicities as compared to monospecific ADCs.

### 3.1. BsADC Design and Payload Considerations

The therapeutic efficacy and tolerability of bsADCs is highly dependent on the choice of linker and payload, as well as the conjugation approach [37]. To enable safe and effective development of bsADCs, linker design requires balancing circulation stability with efficient release of payload at the target site. Linkers are typically categorized as cleavable or non-cleavable, with peptide-cleavable formats dominating the clinical treatment landscape for CRC [38]. Cleavable linkers are designed to rapidly release payloads under precise physiological conditions within the tumor microenvironment or intracellularly. Further, they may improve efficacy in heterogeneous tumors by eliciting a bystander effect, which allows payload released from target cells to destroy neighboring cells. The pH-sensitive linkers exploit the acidic environment of the endosomes and lysosomes, and glutathione-sensitive linkers capitalize on the highly reducing cytosolic compartment. The most common cleavable designs are protease-sensitive peptide linkers, which are largely cleaved by lysosomal enzymes (i.e., cathepsin B). In bsADCs currently undergoing clinical investigation for CRC, linkers are largely proprietary or not publicly disclosed, but likely incorporate peptide linkers such as the reliable cathepsin-sensitive dipeptide valine-citrulline (VC), less common tripeptides like valine-lysine-glycine (VKG), or the tetrapeptide sequence glycine-glycine-phenylalanine-glycine (GGFG) [39]. Non-cleavable linkers require complete proteolytic degradation of the antibody for payload release, providing greater stability at the cost of relying on total intracellular processing for activity. In addition, conjugation strategies, including site-specific approaches (e.g., engineered cysteine-, glutamine-, or glycan-based conjugation) and conventional stochastic lysine- or cysteine-based conjugation, influence batch homogeneity, drug-to-antibody ratio (DAR), and the therapeutic index of these molecules [40].

Commonly utilized ADC payloads include topoisomerase I inhibitors (Top1i; e.g., deruxtecan [DXd], exatecan derivatives), microtubule inhibitors (e.g., monomethyl auristatin E [MMAE], monomethyl auristatin F [MMAF]), and DNA-damaging agents (e.g., calicheamicin and pyrrolobenzodiazepine [PBD] dimers) [15]. Among these, Top1i payloads have emerged as particularly successful and are incorporated into several recently approved ADCs. Top1is have also demonstrated particular success as components of combination chemotherapeutic regimens in CRC, reflecting a compatibility with CRC biology and sensitivity profiles [3,5]. This is further supported by the clinical activity of the Top1i payload-conjugated ADC T-DXd in HER2-positive mCRC, where the DESTINY CRC-001 trial demonstrated a confirmed objective response rate of 45.3% with durable responses and a manageable tolerability profile, ultimately leading to regulatory approval in this setting [41,42,43].

### 3.2. BsADCs Mechanisms of Action

**Figure 3:**
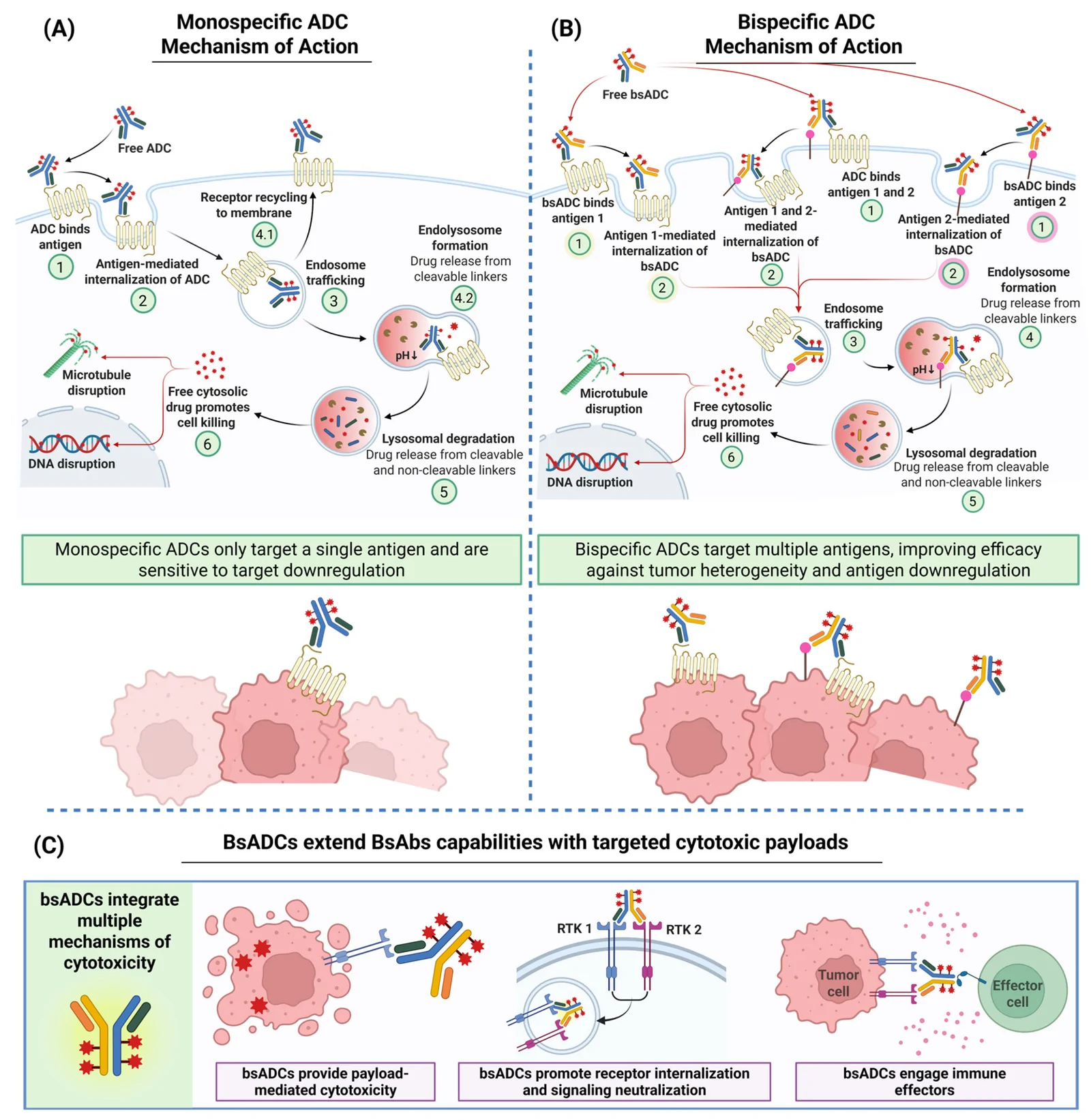
Bispecific antibody–drug conjugate mechanism of action. (**A**,**B**) Monospecific ADCs and BsADCs bind target antigen(s) on the cell surface (1) and internalize (2) to endosomes (3). The endosome can either recycle back to the plasma membrane (4.1) or fuse with a lysosome to create an endolysosome (4.2 and 4). Enzymatic activity and/or low pH within the lysosome prompt ADC/bsADC drug release through linker cleavage and/or degradation (5), resulting in tumor cell-killing (6). Additionally, bsADCs can internalize through binding to either target antigen individually or through bsAb-mediated interaction between both targets. (**C**) BsADCs preserve the native functions of bsAbs, such as receptor internalization, signaling suppression, or immune cell engagement, while enhancing antitumor efficacy through targeted delivery of a cytotoxic payload.

Like monospecific ADCs, bsADCs exert their effects primarily through target-mediated internalization and intracellular payload release (Figure 3A,B). Upon binding to tumor-associated antigens, bsADCs are internalized via endocytosis and trafficked to lysosomes, where linker cleavage or degradation of the bsADC releases the cytotoxic payload, resulting in on-target cell-killing (Figure 3B) [37]. Notably, bsADCs may only need to bind a single antigen to elicit cytotoxic effects. Because cytotoxic payload release occurs downstream of cell surface antigen binding, bsADCs/ADCs can eliminate tumor cells regardless of mutations that may confer resistance to classical mAb or bsAb therapies, such as *KRAS* mutations, which are observed in ~35.9% of mCRCs [2]. Depending on the targets selected, bsADCs can retain bsAb activity, including inhibition of oncogenic signaling pathways and/or generation of an immune response (Figure 3C). Importantly, dual antigen recognition may enhance internalization efficiency or broaden the population of targetable tumor cells compared to monospecific ADCs, improving overall therapeutic efficacy and providing rationale for the pursuit of bsADC development [37].

Through targeting multiple antigens, bsADCs may also overcome loss of target expression that frequently confers resistance to monospecific ADCs. By recognizing two distinct tumor-associated antigens, bsADCs can target a broader and more heterogeneous population of tumor cells, including subpopulations with low or variable expression of either antigen alone, thereby reducing the likelihood of therapeutic escape through antigen downregulation. In addition, the use of highly potent payloads, including Top1is with established activity in CRC, may enable bsADCs to maintain efficacy even in heavily pretreated or refractory tumors.

## 4. Clinical Status of bsAbs in Colorectal Cancer

Although bsAbs have transformed the treatment landscape for several hematologic malignancies and are increasingly being approved for solid tumors, clinical translation in CRC remains in its early stages. At present, limited peer-reviewed safety and efficacy data have been formally published for many ongoing studies, reflecting their early-phase status. Nevertheless, several classes of bsAbs, including immune T cell engagers, dual checkpoint inhibitors, dual signaling pathway inhibitors, and biparatopic antibodies, have demonstrated encouraging early clinical activity and continue to expand the therapeutic landscape for CRC. A summary of current clinical-stage bsAbs being evaluated in CRCs is shown in Table 1.

### 4.1. Immune Cell Engagers

Early clinical development of bsAbs for CRC largely centered on T cell redirection through CD3 engagement, exemplified by the EpCAM × CD3 trifunctional antibody catumaxomab and CEA × CD3-engaging MEDI-565 and cibisatamab. Catumaxomab, which received European approval in 2009 for the treatment of malignant ascites associated with EpCAM-positive carcinomas [44,45,46], simultaneously engages EpCAM-expressing tumor cells, CD3-positive T cells, and FcγR-expressing immune cells through its hybrid mouse/rat IgG1, promoting coordinated antitumor immunity [44,47]. In preclinical CRC models, catumaxomab demonstrated substantially greater tumor cell killing than parental mAbs through enhanced immune-mediated cytotoxicity. However, the enhanced Fc function was ultimately one of the limitations of this modality, as it may mediate CD3-positive, EpCAM-negative cell killing, and clinical use was frequently associated with cytokine-related toxicities and hepatotoxicity. The drug was voluntarily withdrawn from the European market in 2017. These limitations highlighted the importance of tumor-selective target expression and motivated development of next-generation bsAbs with improved pharmacologic properties and better control of immune activation. Currently, most CD3-directed bsAbs incorporate mutations in the Fc region of IgG1 to silence Fc-mediated immune activation, or use IgG4, which has heavily reduced effector functions. More recently, additional CD3-directed bsAbs targeting CDH17 (ARB202; NCT05411133), B7-H3/CD276 (NCT05999396), and nectin-4 (NCT07545122) entered early-phase clinical evaluation. These agents are generally being evaluated in patients with advanced or mCRC regardless of MSI status, although enrollment is frequently enriched for tumors expressing the target antigen and predominantly MSS disease, where effective immunotherapies remain limited.

B7-H3 (CD276) has also been identified as a promising immune-engaging target because of its elevated expression in CRC relative to most normal tissues [48,49]. Although its physiological function has not been fully elucidated, B7-H3 is a member of the B7 immune checkpoint family that is thought to negatively regulate T cell activation and effector function, while its overexpression in many solid tumors has been associated with immune evasion, tumor progression, metastasis, and poor clinical outcomes [50]. A recently developed IgG-like B7-H3 × CD3 bsAb demonstrated potent T cell activation and antigen-dependent cytotoxicity across multiple CRC cell lines while producing tumor regression in xenograft models [51]. Importantly, this bsAb incorporates an affinity-attenuated CD3-binding arm that reduces nonspecific T cell activation while maintaining high-avidity binding to B7-H3, resulting in substantially lower cytokine release than conventional CD3-engaging antibodies and suggesting that optimized molecular architecture and affinity tuning may improve therapeutic index [51]. This strategy has now advanced into early-stage clinical evaluation (NCT05999396), with data pending.

Beyond CD3-directed T cell engagers, immune-modulatory bsAbs targeting immune checkpoints and angiogenic pathways have quickly become an active area of clinical development in CRC. Dual PD-1/PD-L1 × VEGF blockade is designed to simultaneously relieve T cell suppression while normalizing the tumor vasculature, promoting immune cell infiltration and enhancing antitumor immunity [52]. Ivonescimab (PD-1 × VEGF) has demonstrated encouraging clinical activity with a manageable safety profile across multiple solid tumors and is currently being evaluated in a phase II trial for previously treated mCRC (NCT06959550). Similarly, PF-08634404 (PD-1 × VEGF) has shown early antitumor activity and tolerability in phase I studies warranting advancement into a global phase III trial in combination with chemotherapy versus bevacizumab plus chemotherapy as first-line treatment for mCRC (NCT07222800). Additional immune-engaging strategies seek to locally activate costimulatory receptors within the tumor microenvironment. For example, givastomig (CLDN18.2 × 4-1BB) is designed to spatially restrict immune activation to CLDN18.2-positive tumors, enhancing antitumor immunity while minimizing systemic hepatotoxicity associated with earlier 4-1BB agonists [53].

### 4.2. Dual Checkpoint Inhibitors

Dual immune checkpoint-targeting bsAbs aim to improve upon conventional PD-1/CTLA-4-targeting mAb combination therapy while reducing systemic immune-related toxicities through coordinated checkpoint blockade [54,55]. Building on preclinical evidence that dual checkpoint blockade can enhance T cell activation and clinical activity in other tumor types, next-generation checkpoint-targeting bsAbs are now being evaluated clinically in CRC. Agents including cadonilimab (AK104; PD-1 × CTLA-4), KN046 (PD-L1 × CTLA-4), and SI-B003 (PD-1 × CTLA-4) are undergoing clinical evaluation in advanced CRC (e.g., NCT04556253, NCT04606472, NCT05985109), with many studies focused on patients with MSS disease or those refractory to prior immune checkpoint inhibitors. More recently, AK129 (PD-1 × LAG-3; NCT06943820) has entered early-phase clinical evaluation after showing favorable anti-tumor activity in mouse models of CRC tumors, reflecting growing interest in simultaneously targeting multiple inhibitory pathways to overcome adaptive immune resistance [56].

### 4.3. Dual Signaling Pathway Inhibitors

Several bsAbs have also been developed to simultaneously inhibit complementary oncogenic signaling pathways. Dual-targeting strategies commonly focus on RTKs and associated pathways, including EGFR, HER2, HER3, and c-MET, which regulate mitogen-activated protein kinase (MAPK) and PI3K/AKT signaling and frequently contribute to therapeutic resistance in CRC [18]. Among these, amivantamab (EGFR × c-MET) is the most clinically advanced and has received regulatory approval for EGFR-altered non-small cell lung cancer. Mechanistically, amivantamab promotes receptor clustering, internalization, and degradation while simultaneously blocking ligand binding and c-MET-mediated bypass signaling, an adaptive resistance mechanism where tumor cells utilize c-MET activation to maintain downstream proliferative and survival pathways despite EGFR blockade [32,57]. Additionally, amivantamab retains normal IgG1 Fc immune-activating function and may activate ADCC or ADCP. In mCRC, amivantamab is being evaluated in patients with *RAS/BRAF* wild-type disease. Results from the phase Ib/II OrigAMI-1 study demonstrated objective response rates of 19–29% across molecularly defined patient cohorts, with median durations of response ranging from 6.1 to 9.8 months. Grade ≥ 3 treatment-related adverse events (TRAEs) were infrequent, most commonly rash (7%), dermatitis acneiform (4%), and hypoalbuminemia (4%), and only one patient discontinued treatment because of a TRAE, supporting continued clinical development [58]. Building on these findings, amivantamab plus chemotherapy is now being evaluated in two randomized phase III trials: first-line treatment in combination with modified FOLFOX6 (OrigAMI-2) and second-line treatment in combination with FOLFIRI (OrigAMI-3) (NCT06750094), with the goal of improving outcomes by simultaneously inhibiting EGFR signaling and c-MET-mediated resistance pathways. Another EGFR × c-MET bsAb strategy is being pursued with MCLA-129, an IgG1-based bsAb designed to inhibit EGFR signaling while blocking c-MET-mediated compensatory activation [59]. MCLA-129 also incorporates reduced fucosylation in the Fc region of its wild-type IgG1 structure, enhancing ADCC function [60]. Although both amivantamab and MCLA-129 target the same receptor pair, differences in antibody engineering, epitope selection, and receptor engagement properties may influence receptor internalization, pathway inhibition, and clinical activity. MCLA-129 has demonstrated preclinical antitumor activity in EGFR/c-MET-dependent models and is under clinical evaluation in advanced solid tumors, including CRC (NCT04930432). Similarly, the IgG1-like EGFR × HER3 bsAb SI-B001 (izalontamab) was developed to suppress HER3-mediated compensatory signaling that frequently emerges following EGFR inhibition. In a first-in-human phase I/Ib study involving patients with advanced epithelial tumors, SI-B001 achieved a disease control rate of 37% with two confirmed partial responses and stable disease in 18 patients, including one patient with mCRC [61]. TRAEs were predominantly grade 1–2; most commonly rash, infusion-related reactions, and paronychia, with no dose-limiting toxicities or treatment-related deaths observed. Based on the overall safety, pharmacokinetic, and preliminary efficacy data, a recommended phase II dose of 9–16 mg/kg administered weekly was established. Although CRC-specific efficacy data remain limited, the single enrolled patient with mCRC achieved stable disease, warranting continued evaluation of SI-B001 in CRC and other epithelial malignancies.

### 4.4. CSC-Targeting Modalities

Outside of classical receptor tyrosine kinases, novel CRC-specific targets continue to emerge. LGR5 is a marker of both normal ISCs and colorectal CSCs that has important functions in tumor initiation, metastasis, and therapeutic resistance [62]. Petosemtamab (MCLA-158), an IgG1-like bsAb targeting EGFR × LGR5, is currently being evaluated in MSS, *RAS/RAF* wild-type mCRC as a monotherapy in the third-line or later setting and in combination with FOLFOX or FOLFIRI as first- or second-line therapy in EGFR therapy-naïve patients (NCT03526835) [63,64]. Similar to MCLA-129, petosemtamab also incorporates low levels of fucose in its modified Fc region, which likely functions to enhance ADCC and contribute to enhanced efficacy in combination with immune checkpoint blockade, which is being evaluated in clinical trials for head and neck cancer (NCT03526835, NCT06525220) [65,66]. Mechanistically, petosemtamab simultaneously inhibits EGFR signaling and degrades EGFR via constitutive LGR5-mediated internalization [36,62,64,67], representing an obligate mechanism of action that distinguishes petosemtamab from conventional EGFR-targeting mAbs. Importantly, petosemtamab demonstrated selective activity in CRC tumor-derived organoids over normal intestinal-derived organoids, whereas EGFR mAbs exhibited identical cytotoxicity in normal and tumor-derived models [64]. These findings indicate that the overexpression of LGR5 in CRC likely functions to enhance tumor selectivity compared to traditional EGFR-targeting mAbs. Further, EGFR inhibition has been shown to increase LGR5 expression in several models [17,36,68,69], which may contribute to enhanced petosemtamab activity in EGFR mAb-refractory CRC tumors. Phase II petosemtamab data demonstrated antitumor activity across early- and late-line treatment settings. In first-line EGFR therapy-naïve patients receiving petosemtamab plus chemotherapy, partial responses were observed, including 3/3 evaluable patients in the initial first-line cohort. In the second-line cohort, 4/8 evaluable patients achieved partial responses (including two unconfirmed responses) [63]. In the third-line or later monotherapy cohort, 1/14 evaluable patients achieved a partial response and 6/14 achieved stable disease, suggesting durable disease control in a subset of heavily pretreated patients. TRAEs were generally consistent with EGFR pathway inhibition and were predominantly low grade, with rash, infusion-related reactions, and gastrointestinal toxicities among the most commonly reported events, supporting continued clinical development.

### 4.5. Biparatopic Antibodies

HER2-targeting biparatopic antibodies, including zanidatamab and KN026, represent another important class of signaling-directed bsAbs [20,70]. Both are IgG1-like biparatopic antibodies that simultaneously bind two distinct, non-overlapping HER2 epitopes within a single molecule, promoting enhanced receptor clustering, internalization, and degradation compared with conventional HER2 mAbs. Clinically, zanidatamab has demonstrated durable responses with a manageable safety profile across HER2-positive malignancies, with diarrhea and infusion-related reactions representing the most common TRAEs, and is currently undergoing clinical evaluation in HER2-amplified mCRC [71]. Additional studies combine zanidatamab with chemotherapy or checkpoint blockade in gastrointestinal cancers (NCT03929666). KN026 has likewise demonstrated favorable safety and preliminary antitumor activity in early-phase studies of HER2-positive solid tumors (NCT05985707), supporting continued investigation in HER2-driven gastrointestinal malignancies, including CRC. Although HER2 amplification occurs in only ~4% of CRC patients, it represents a clinically actionable population that has already benefited from HER2-directed mAbs, tyrosine kinase inhibitors, and ADCs, supporting continued development of biparatopic HER2-targeting strategies.

Collectively, these clinical-stage bsAbs illustrate the rapid diversification of bsAb development in CRC beyond conventional CD3-directed T cell engagers. Current efforts increasingly combine immune engagement, checkpoint blockade, angiogenesis inhibition, costimulatory signaling, or dual receptor inhibition within a single molecule to improve activity against predominantly MSS CRC while minimizing cytokine release syndrome, immune-related adverse events, and on-target/off-tumor toxicity.

## 5. Clinical Status of bsADCs in Colorectal Cancer

BsADCs combine the dual-targeting capabilities of bsAbs with the potent cytotoxic activity of ADC payloads, enabling selective delivery of chemotherapy while simultaneously engaging two tumor-associated antigens or epitopes. This design has the potential to improve tumor selectivity, enhance internalization, increase tumor coverage, and reduce antigen escape compared with conventional monospecific ADCs. Despite these promising advantages, no bsADC has yet received regulatory approval in the United States for any indication, although izalontamab brengitecan has recently been approved in China. Nevertheless, the field is advancing rapidly, with several bsADC candidates entering early-phase clinical trials for solid tumors, including CRC. A summary of current clinical-stage bsADCs being evaluated in CRCs is shown in Table 2.

### 5.1. EGFR × HER3 bsADCs

The most clinically advanced bsADC is izalontamab brengitecan (BL-B01D1), an IgG-like EGFR × HER3 bsADC that incorporates a Top1i payload (Ed-04; DAR = 8) through a cleavable linker. Preclinical studies demonstrated robust antitumor activity in EGFR/HER3-expressing colorectal and pancreatic cancer xenograft models, consistent with enhanced dual-target engagement, receptor internalization, and delivery of the Top1i payload [72]. In a first-in-human phase I study of BL-B01D1 in advanced solid tumors, the agent demonstrated preliminary antitumor activity, particularly in EGFR-expressing malignancies, while establishing a recommended phase II dose of 2.5 mg/kg on days 1 and 8 every 3 weeks. TRAEs were primarily hematologic, including neutropenia (47%), anemia (39%), leukopenia (39%), and thrombocytopenia (32%), with grade ≥ 3 TRAEs occurring in 71% of patients; however, treatment discontinuation due to toxicity was uncommon (3%) [73]. In trials focused on EGFR-mutant non-small cell lung cancer, izalontamab brengitecan demonstrated encouraging clinical activity with a manageable safety profile, providing clinical validation for EGFR × HER3 bsADCs [74]. Clinical studies are currently evaluating its therapeutic potential in gastrointestinal malignancies, including CRC (NCT06008054).

The clinical development of EGFR × HER3 bsADCs has expanded beyond izalontamab brengitecan to include several next-generation agents. JS212, an EGFR × HER3 bsADC conjugated to a Top1i payload (exatecan, DAR = 6), recently entered first-in-human phase I evaluation in patients with advanced solid tumors, including gastrointestinal malignancies (NCT06888830) [75]. Although clinical efficacy data have not yet been published, preclinical studies demonstrated potent antitumor activity across multiple EGFR- and HER3-expressing tumor models, favorable pharmacokinetic properties, and acceptable safety in non-human primates, supporting continued clinical development [76]. Similarly, additional EGFR × HER3 bsADCs, including DB-1418 (P1021 payload, proprietary Top1i; DAR = 6), are undergoing early clinical evaluation for advanced solid tumors, further reflecting growing interest in dual targeting EGFR and HER3 as a strategy to enhance tumor selectivity and potentially address compensatory signaling pathways [77].

### 5.2. EGFR × c-MET bsADCs

DM005 is an EGFR × c-MET bsADC conjugated to the Top1i BCPT02 (DAR ~ 4) that recently entered first-in-human phase I clinical evaluation in patients with advanced solid tumors, including mCRC (NCT06515990). Preclinical characterization demonstrated potent, dose-dependent antitumor activity in both cell line-derived and patient-derived xenograft models, efficient dual-target-mediated internalization, and favorable pharmacokinetics and tolerability in non-human primates, supporting further clinical development [78]. The ongoing phase I study is enrolling patients with advanced CRC and other EGFR- and/or c-MET-expressing solid tumors to evaluate safety, pharmacokinetics, and preliminary antitumor activity, with dose escalation followed by expansion cohorts in selected tumor types [79]. Although CRC-specific efficacy data have not yet been reported, the inclusion of dedicated CRC cohorts highlights the growing clinical interest in EGFR × c-MET-targeted bsADCs for overcoming resistance to EGFR-directed therapies.

The EGFR × c-MET bsADC AZD9592 demonstrated dose-dependent antitumor activity across a panel of CRC patient-derived xenograft models, with responses correlating with c-MET expression and extending to *KRAS*- and *BRAF*-mutant tumors [80,81]. Incorporating a cleavable linker and Top1i payload, AZD9592 exhibited favorable preclinical tolerability and has subsequently advanced to early-phase clinical evaluation (NCT05647122), supporting the feasibility of dual-targeted payload delivery for CRC [80].

### 5.3. PD-L1 × VEGFR2 bsADC

An additional advancement in bsADC design is the concept of immune-cytotoxic convergence, in which a single molecule combines immune modulation with targeted payload delivery. JSKN027, a bispecific immunomodulatory ADC targeting PD-L1 and VEGFR2 (NCT07391644), is designed to simultaneously enhance T cell activation while delivering a cytotoxic payload to tumor cells (payload and DAR have not been publicly disclosed). Clinical evaluation is soon to be initiated. This strategy exemplifies the growing interest in multifunctional biologics capable of simultaneously targeting tumor-intrinsic signaling and the tumor microenvironment.

### 5.4. Biparatopic HER2 bsADCs

Biparatopic HER2-targeting bsADCs have also entered clinical evaluation for HER2-positive mCRC. JSKN003, a biparatopic HER2 ADC incorporating a Top1i payload (DXd; DAR ~ 4), is currently being evaluated in a phase I/II trial enrolling patients with advanced HER2-expressing solid tumors, including mCRC (NCT05744427) [82,83]. Early clinical data have demonstrated encouraging antitumor activity across HER2-positive malignancies with a manageable safety profile, supporting continued clinical development. Similarly, zanidatamab zovodotin (ZW49), an ADC derived from the biparatopic HER2 bsAb zanidatamab and conjugated to the auristatin F derivative AF-HPA payload (DAR ~ 2), demonstrated preliminary clinical activity in HER2-expressing solid tumors, including gastrointestinal cancers, with a safety profile consistent with auristatin-based ADCs [84]. These studies highlight the continued clinical interest in biparatopic HER2-directed bsADCs for patients with HER2-amplified CRC.

Collectively, bsADCs represent rapidly evolving therapeutic modalities with substantial potential to improve treatment outcomes in CRC. Although most clinical candidates remain in early-phase development, advances in antibody engineering, target selection, and payload conjugation continue to improve efficacy while reducing toxicity. As additional safety and efficacy data emerge from ongoing clinical trials, these agents are expected to play an increasingly important role in precision therapy for CRC.

## 6. Emerging Preclinical Bispecific Modalities and Strategies to Enhance Efficacy in Colorectal Cancer

The development of next-generation targeted therapies for CRC requires preclinical strategies that address the complex biological and immunological barriers underlying treatment resistance. Key challenges, including low intrinsic immunogenicity, limited T cell infiltration, substantial tumor antigen heterogeneity, and the emergence of adaptive resistance pathways necessitate optimization of target selection, molecular architecture, immune engagement, and therapeutic payload delivery strategies for bsAbs and bsADCs [6,85,86,87]. In addition to enhancing tumor specificity and immune activation, emerging bispecific platforms aim to improve therapeutic selectivity by simultaneously targeting complementary tumor vulnerabilities, overcoming antigen escape, and maximizing antitumor activity while minimizing on-target/off-tumor toxicity. For bsAbs, these efforts have focused on expanding targetable antigen classes, remodeling the immunosuppressive tumor microenvironment, and engineering improved safety profiles through conditional activation and optimized immune engagement. For bsADCs, bispecific designs provide opportunities to enhance tumor coverage, facilitate receptor-mediated internalization, and deliver cytotoxic payloads to heterogeneous tumor populations. Collectively, these preclinical strategies seek to achieve a balance between potent tumor killing, durable therapeutic responses, and an improved therapeutic window [19,88].

### 6.1. Preclinical bsAbs

Beyond conventional cell-surface targets, next-generation bsAbs are expanding the therapeutic landscape in CRC through novel antigen recognition and improved safety engineering strategies. T cell receptor (TCR)-mimic bsAbs targeting intracellular neoantigens presented by human leukocyte antigen (HLA) molecules, including KRAS^G12V^- and KRAS^G12D^-derived peptides, have demonstrated selective killing of mutant CRC cells, enabling therapeutic targeting of intracellular alterations past traditional surface antigens [89]. In parallel, immunomodulatory bsAbs are being developed to remodel the immunosuppressive CRC tumor microenvironment. For example, the PD-L1 × tumor necrosis factor receptor 2 (TNFR2) bsAb ATAPL1 demonstrated enhanced tumor accumulation and antitumor activity compared with single-agent blockade in preclinical CRC models by simultaneously inhibiting PD-L1 signaling and reducing immunosuppressive cell populations, including regulatory T cells and monocytic myeloid-derived suppressor cells, while enhancing CD8^+^ T cell activation and improving responses to chemotherapy [90]. To improve the therapeutic window of immune-engaging approaches, additional engineering strategies include conditionally active biological (CAB) bsAbs, which restrict immune activation to the tumor microenvironment through tumor-specific features such as acidic pH. For example, CAB bsAbs targeting lymphocyte antigen 6 family member G6D (LY6G6D) × CD3, CEACAM5 × CD47, CD47 × PD-L1, or c-MET × PD-L1 have been explored to enhance selectivity and minimize toxicity. Additional optimization of CD3-binding affinity and Fc engineering has further improved safety profiles by reducing excessive cytokine release and FcγR-mediated immune activation. Affinity-tuned CD3 engagement can preserve antitumor activity while limiting cytokine production, whereas Fc silencing or Fc removal reduces off-target immune interactions, although these modifications require balancing safety improvements with antibody stability and pharmacokinetic properties.

### 6.2. Preclinical bsADCs

While advances in bsAb engineering have expanded therapeutic opportunities in CRC, several barriers continue to limit clinical translation, including systemic immune toxicity, limited T cell infiltration, and an immunosuppressive tumor microenvironment [6]. These challenges have driven interest in bsADCs. Emerging preclinical bsADC platforms in CRC include CDH17 × guanylate cyclase 2C (GUCY2C) bsADC conjugated with ferroptosis inducer RAS-selective lethal 3 (RSL3) payloads (DAR = 4) [91], EGFR × ephrin type-A receptor 2 (EphA-2) PBS293 with MMAE (DAR = 4), CDH17 × CEA bsADC GS24-B025 with undisclosed Top1i (DAR = 8) [92], and CEACAM5 × EGFR bsADC RT023 with Top1i (exatecan, DAR = 6) [93]. By simultaneously targeting complementary tumor antigens, these approaches aim to improve tumor coverage, enhance payload delivery, and mitigate antigen-negative escape. Preclinical studies have demonstrated potent anti-tumor activity across CRC cell lines, organoids, and xenograft models, supporting further development of this approach.

### 6.3. Preclinical CSC-Targeting bsAbs and bsADCs

Perhaps one of the largest barriers to therapeutic targeting of CSCs in CRC thus far is the mitigation of on-target, off-tumor toxicities. Bispecific approaches that target CSC markers and CRC-enriched cell surface antigens may therefore offer a compelling approach to target CSCs while enhancing tumor specificity. Petosemtamab, which demonstrates improved tolerability and efficacy over EGFR mAbs in pre-clinical studies, likely due to LGR5 overexpression in CRC, has advanced further in clinical trials than the previously reported LGR5 mAb BNC101. This supports the notion that bsAbs are better equipped to target CSCs than monospecific approaches. While LGR5 remains the only CSC marker under active clinical investigation, EpCAM-targeting bsAbs have shown promise in preclinical CRC models. For example, a CD3 × EpCAM CAB bsAb, which displays improved binding to CD3 and EpCAM under tumor-associated acidic pH, demonstrated similar antitumor efficacy to a non-CAB CD3 × EpCAM bsAb while reducing systemic immune activation and improving tolerability in preclinical models [94]. These studies support future efforts to develop bsAbs targeting other CSC markers, harnessing either tumor microenvironment properties or CSC marker overexpression to achieve tumor selectivity.

The same principles described above for CSC-targeting bsAbs may reasonably be applied to bsADCs. As mentioned for petosemtamab, EGFR × LGR5 represents a particularly promising strategy for addressing CRC heterogeneity and cancer stem cell-associated resistance [95]. An EGFR × LGR5 bsADC conjugated to a Top1i (camptothecin 2 (CPT2); DAR = 8) demonstrated enhanced cytotoxicity compared to LGR5 ADC and EGFR × HER3 bsADC izalontamab brengitecan, with activity dependent on dual antigen engagement and receptor-mediated internalization [36]. In patient-derived xenograft models, EGFR × LGR5 bsADC induced tumor regression and outperformed EGFR mAb, EGFR × LGR5 bsAb, and LGR5 ADC therapies, supporting the potential of dual-targeted bsADCs to overcome tumor heterogeneity and CSC-driven resistance [36]. In line with these findings, EpCAM-directed bsADCs co-targeting HER3 (BCG044) with Top1i (BLD1102, DAR undisclosed) [96] or CLDN3 with Top1i (Dxd, DAR = 4) [97] have also been reported. In CRC patient-derived xenografts, BCG044 exerted comparable antitumor efficacy to parental EpCAM ADC and superior efficacy over HER3 parental ADC [96]. Importantly, BCG044 demonstrated superior tolerability over both parental ADCs in humanized mice, indicating that this bispecific modality confers improved tumor selectivity over monospecific ADCs [96]. Similarly, EpCAM x CLDN3 bsADC demonstrated strong binding and antitumor activity in EpCAM- and CLDN3-high models, with weak binding and reduced antitumor activity in EpCAM-high and CLDN3-low models [97]. These findings highlight bsADCs as a promising strategy to improve therapeutic selectivity and tolerability for CSCs in CRC, although further clinical validation is required to support this approach.

## 7. Limitations and Challenges of Bispecific Approaches in CRC

While bispecific modalities demonstrate significant potential for treating CRC, several limitations and challenges remain. Particularly relevant with immune-engaging bsAbs, achieving the appropriate levels of immune activation to maximize immune-mediated cytotoxicity while minimizing overactivation and serious immune-related adverse events remains a major difficulty. Further, it is likely that different bsAb antigen pairs will require different approaches to modulate immune activation. For example, catumaxomab retains a highly active hybrid mouse/rat Fc region [98], which may in part have contributed to its serious immune-related adverse events. Accordingly, approaches such as CAB bsAbs, Fc-null bsAbs, or bsAbs entirely lacking an Fc have been employed to improve immune-related safety profiles of other immune-engaging bsAbs [99]. Conversely, immune-activating Fc engineering, such as that observed with petosemtamab [95], may also be employed. However, this approach is largely reserved for bsAbs that do not primarily function through immune cell engagement. Importantly, Fc engineering to enhance immune-related functions may be critical to improve patient responses in MSS CRC, particularly in combination with immune checkpoint inhibitors, though clinical validation of this approach is still needed.

Another difficulty that antibody-based therapeutics often encounter is limited tumor penetration due to slow-internalizing target antigens or the binding-site barrier wherein antibodies or ADCs primarily bind to the periphery of tumors [100]. Approaches including antibody co-administration with ADCs to saturate tumor periphery binding or ADC co-administration with high avidity, low affinity antibodies have been shown to improve ADC tumor penetration and reduce on-target, off-tumor toxicities in antigen-high and antigen-low settings [101], though these approaches have yet to be explored with bsAbs and bsADCs. Importantly, the design of bsAbs or bsADCs that target a constitutively internalizing receptor, such as LGR5, with one arm may function to improve tumor penetration, though more detailed mechanistic preclinical and clinical studies are necessary to validate this idea.

Lastly, balancing bsAb and bsADC efficacy with toxicity profiles will be paramount to their clinical success. Encouragingly, the tolerability profiles of clinical-stage bsAbs have been largely manageable, with several of these agents (e.g., petosemtamab, givastomig, CC-3) reporting reduced toxicity compared to their mono-targeting counterparts. The improved tolerability of these agents is likely due to the CRC overexpression of LGR5, CLDN18.2, and B7-H3, respectively. Furthermore, as previously discussed, Fc engineering or removal has been explored extensively to improve the immune-related adverse event profile of immune-engaging bsAbs. Regarding bsADCs, clinical readouts indicate that tolerability profiles are largely aligned with that of their associated payload class, as is observed for monospecific ADCs. Comprehensive preclinical mechanistic tolerability studies and the continued progression of bsAbs and bsADCs in CRC trials will be critical to continue to elucidate the tolerability profiles of these agents.

## 8. Concluding Remarks and Future Directions

BsAbs and bsADCs represent rapidly expanding therapeutic modalities that have the potential to significantly reshape the treatment landscape of CRC. Across preclinical and early clinical development, these platforms are demonstrating strong antitumor activity through dual-target engagement, immune cell redirection, enhanced tumor selectivity, and targeted cytotoxic payload delivery, offering clear advantages over monospecific antibody approaches in a disease characterized by marked heterogeneity and adaptive resistance. By combining dual-antigen recognition with potent cytotoxic payloads, bsADCs are particularly well suited to overcome tumor heterogeneity, antigen loss, and resistance mechanisms while maintaining the precision of antibody-based targeting.

Although an increasing number of bsAbs have entered clinical evaluation for CRC, most bsADCs are largely confined to preclinical development or first-in-human clinical trials. Early clinical programs evaluating agents such as izalontamab brengitecan (EGFR × HER3), DM005 (EGFR × c-MET), JSKN003 (biparatopic HER2), and other next-generation bsADCs have demonstrated encouraging preliminary safety and antitumor activity across HER3-, HER2-, EGFR-, and c-MET-expressing solid tumors, providing important proof-of-concept for dual-targeted payload delivery and supporting continued investigation in mCRC (NCT06008054) [79,82]. This contrasts with the more established success of bsAbs in hematologic malignancies and illustrates the persistent challenges associated with solid tumors, including limited immune cell infiltration, antigen heterogeneity, inefficient intratumoral drug delivery, and the immunosuppressive tumor microenvironment [6,29,31]. To date, these barriers have constrained the number of bispecific therapies achieving regulatory approval or broad clinical impact in solid cancers, despite substantial progress in molecular engineering.

Looking forward, continued advances in antibody engineering and conjugation technologies are likely to expand bsAbs and bsADCs beyond conventional dual-targeting and cytotoxic mechanisms. One emerging direction is the integration of antibody targeting with therapeutic nucleic acids. Antibody–oligonucleotide conjugates can facilitate cell- and tissue-selective delivery of antisense oligonucleotides, siRNAs, and other RNA-modulating agents, potentially expanding bsAb-based therapy to intracellular and otherwise difficult-to-drug targets [102]. Further, bsAbs may be conjugated with nucleic acids modified with boron clusters or radioisotopes (i.e., Lutetium-177) and combined with radiotherapy to enhance antitumor efficacy [103,104]. Although these approaches have not yet been directly integrated into clinically advanced bsAbs or bsADCs for CRC, they illustrate the potential for future multi-specific platforms that combine extracellular antigen recognition with gene regulation or radiotherapy. Additional emerging strategies include next-generation bsADCs incorporating alternative payload classes, immune-stimulatory agents, or even dual payloads with complementary mechanisms of action. Collectively, these advances suggest that the next generation of bispecific therapeutics may evolve from simple dual-antigen recognition toward multifunctional platforms capable of integrating tumor targeting, intracellular pathway modulation, immune activation, and complementary cytotoxic or radiation-based mechanisms.

Key unanswered questions remain regarding optimal therapeutic positioning and combination strategies for bsAbs and bsADCs in CRC. It is not yet clear whether early use of T cell-engaging bsAbs may contribute to T cell exhaustion or alter responsiveness to subsequent immunotherapies, and the ideal integration of bsAbs with existing standards of care has not yet been fully defined. Similarly, the most effective sequencing of bsADCs with immunotherapy, chemotherapy, or radiotherapy remains an open area of investigation. A particularly promising strategy is the rational combination of bsADCs and bsAbs, in which cytotoxic tumor debulking by bsADCs may be followed by immune activation via bsAbs to sustain longer-term antitumor responses and reduce relapse driven by residual resistant clones [31,35]. Future progress will likely depend on continued innovation in antibody engineering, including multi-specific formats, improved linker-payload optimization, novel payloads, conditionally active and tumor-selective designs, and more precise tumor-selective targeting strategies capable of overcoming antigen escape and intratumoral heterogeneity. Further, target discovery combined with advances in preclinical modeling systems such as tumor organoids and immune-reconstituted models will be critical for improving translational predictability. As our understanding of CRC biology continues to expand, bsAbs and bsADCs are well positioned to address important unmet clinical needs and are likely to play a central role in the next generation of precision cancer therapeutics.

## Figures and Tables

**Table 1:** Clinical stage bsAbs.

Bispecific Antibody	Targets	Clinical Phase (CRC)	Representative CRC Trial(s)	Tolerability	Fc Type	Company	Current Status/Key Findings
Immune cell engager							
Ivonescimab (AK112)	PD-1 × VEGF	III II II II II II II II II II I/II I	NCT07228832 NCT07021950 NCT06959550 NCT06848842 NCT07359456 NCT06790212 NCT06760520 NCT07162714 NCT06718543 NCT06919510 NCT07397338 NCT07363408	Primarily grade 1–2 TRAEs; most common were hypertension and proteinuria consistent with VEGF inhibition. No new safety signals identified.	Mutated IgG1	Summit/Akeso	Being evaluated in combination with chemotherapy, radiotherapy, or RAS (ON) inhibitors. Being evaluated in metastatic and locally advanced CRC.
PF-08634404 (SSGJ-707)	PD-1 × VEGF	III II I/II	NCT07222800 NCT06493760 NCT05846867	Phase II study in CRC demonstrated a manageable safety profile but detailed clinical safety data are not available.	IgG4	3SBio/Sunshine Guojian /Pfizer	Being evaluated in combination with chemotherapy in mCRC without prior systemic treatment.
Givastomig (TJ033721, ABL111)	CLDN18.2 × 4-1BB	I	NCT04900818	No dose-limiting toxicities up to 18 mg/kg; grade ≥ 3 TRAEs reported in 21% of patients. Toxicity normally associated with 4-1BB activation was reduced.	Mutated IgG1	ABL Bio/NovaBridge	CLDN18.2 expression is rare in CRC (~2%), but significantly higher in other GI cancers; CRC tumor type comprises 4 of 75 enrolled patients.
CT-202 (BA3362)	Nectin-4 × CD3	I	NCT07545122	Clinical safety data are not yet available; GLP toxicology studies show no adverse events in any tissues, including skin.	Mutated IgG1	Context Therapeutics/BioAtla	First-in-human study in CRC.
Cabotamig (ARB202)	CDH17 × CD3	I	NCT05411133	Preliminary data indicate manageable cytokine release syndrome, primarily grade 1–2 TRAEs, with no unexpected toxicities.	IgG4	Arbele	First-in-human T cell engager for GI cancers including CRC. Early clinical evaluation is completed.
NILK-2301	CEACAM5 × CD3	I	NCT06663839	First-in-human trial is ongoing; clinical safety data have not yet been reported.	Mutated IgG1	LamKap Bio/Lonza	Being evaluated in low tumor burden mCRC.
CC-3	B7-H3 × CD3	I	NCT05999396	Clinical safety data are not yet available; preclinical studies demonstrated substantially reduced cytokine release compared with conventional CD3-engaging antibodies.	IgG1	University Hospital Tübingen/academic program	First-in-human study in CRC.
Dual checkpoint inhibitor							
Cadonilimab (AK104)	PD-1 × CTLA-4	III III II II II II II I/II	NCT06832917 NCT06566755 NCT05571644 NCT06168786 NCT06455254 NCT07544784 NCT04556253 NCT05426005	Dual checkpoint blockade was associated with fewer grade ≥ 3 immune-related adverse events than conventional PD-1/CTLA-4 combinations, with immune-mediated toxicities remaining manageable.	Mutated IgG1	Akeso	Multiple studies in MSI-H/dMMR and MSS CRC evaluating combinations with chemotherapy, radiotherapy, or anti-angiogenic agents.
KN046	PD-L1 × CTLA-4	II	NCT05985109	Generally well tolerated in early studies, with manageable immune-related adverse events and no unexpected safety signals reported.	IgG1	Jiangsu Alphamab	Being evaluated in combination with regorafenib in MSS mCRC.
SI-B003	PD-L1 × CTLA-4	II	NCT06008054	Clinical safety data are not yet available.	Likely IgG1	Sichuan Baili Pharmaceutical	Being evaluated for safety and efficacy as a monotherapy and in combination with bsADC izalontamab brengitecan.
AK129	PD-1 × LAG-3	I/II	NCT06943820	Clinical safety data are not yet available.	IgG1	Akeso	Being evaluated for safety and efficacy in chemotherapy combinations.
Dual signaling pathway inhibitor							
Amivantamab	EGFR × c-MET	III II II II I/II	NCT06662786 NCT05845450 NCT06855849 NCT06750094 NCT05379595	Infusion-related reactions, rash, and paronychia were the most common adverse events, with relatively low treatment discontinuation rates.	IgG1	Genmab// Janssen Johnson & Johnson/	Being evaluated as monotherapy and in combination with chemotherapy. Neoadjuvant study in molecularly selected resectable CRC.
MCLA-129	EGFR × c-MET	I/II I/II	NCT04868877 NCT04930432	Predominantly grade 1–2 TRAEs and no dose-limiting toxicities reported during dose escalation.	IgG1	Merus/Genmab	Dose-escalation study includes CRC expansion cohorts.
SI-B001	EGFR × HER3	I	NCT04603287	TRAEs were predominantly grade 1–2, with rash and diarrhea most frequently reported and few treatment discontinuations.	IgG1	Sichuan Baili/SystImmune	Early clinical evaluation includes CRC.
CSC-targeting modalities							
Petosemtamab (MCLA-158)	EGFR × LGR5	III I/II	NCT07702032 NCT03526835	TRAEs were primarily grade 1–2, most commonly rash and infusion-related reactions, with limited grade ≥ 3 toxicity.	IgG1	Merus/Genmab	Being evaluated in unresectable and advanced solid tumors including mCRC.
Biparatopic antibodies							
Zanidatamab (ZW25)	HER2 × HER2	II II II	NCT03929666 NCT07405476 NCT06695845	Diarrhea and infusion-related reactions were the most common adverse events, with relatively few grade ≥ 3 treatment-related events.	IgG1-like/engineered IgG1	Zymeworks/Jazz Pharmaceuticals	Being evaluated in HER2-positive CRC.
KN026	HER2 × HER2	II	NCT05985707	Favorable safety profile with predominantly grade 1–2 adverse events and low rates of grade ≥ 3 toxicity in early clinical studies.	IgG1	Jiangsu Alphamab	Being evaluated in HER2-positive CRC ± KN046.

**Table 2:** Clinical stage bsADCs.

Bispecific ADC	Targets	DAR	Linker/Payload	Clinical Phase (CRC)	Representative CRC Trial(s)	Tolerability	Fc Type	Company	Notes
Izalontamab brengitecan (BL-B01D1)	EGFR × HER3	8	Ed-04 (exatecan-derived topoisomerase I inhibitor) conjugated via protease-cleavable linker	II I	NCT06008054 NCT05262491	Toxicities were primarily hematologic (neutropenia, anemia, leukopenia, thrombocytopenia); grade ≥ 3 TRAEs occurred in ~70–71% of patients, but treatment discontinuation was uncommon (1–3%) and no treatment-related deaths were reported.	IgG1	Sichuan Baili/SystImmune/Bristol Myers Squibb	Most clinically advanced bsADC relevant to CRC. Currently enrolling mCRC patients. Phase I/II studies demonstrated encouraging activity across solid tumors.
JS212	EGFR × HER3	6	Exatecan (topoisomerase I inhibitor) conjugated via cleavable linker	II I/II	NCT07503756 NCT06888830	Grade ≥ 3 TRAEs occurred in 22.8% of patients, commonly neutropenia and leukopenia. MTD not reached.	IgG1 (Fc engineering not disclosed)	Shanghai Junshi Biosciences/BioDlink	Phase I/II data for patients with advanced solid tumors, including GI cancer. Phase II currently enrolling for mCRC.
DB-1418 (AVZO-1418)	EGFR × HER3	6	P1021 (proprietary topoisomerase I inhibitor) conjugated via tetrapeptide-based cleavable linker	I/II	NCT07038343	Clinical safety data are not yet available; demonstrated tolerable safety profile in cynomolgus monkeys with no mortality.	Engineered IgG1; Knobs-into-holes Fc	DualityBio/Avenzo Therapeutics	First-in-human study includes dose escalation and dose expansion phases as single agent or in combination.
AZD9592	EGFR × c-MET	6	Samrotecan (camptothecin-based topoisomerase I inhibitor) conjugated via cleavable linker	I	NCT05647122	Clinical safety data are not yet available.	Engineered IgG1	AstraZeneca	Active, not-enrolling first-in-human study for treatment of multiple advanced solid tumors, including CRC.
DM005	EGFR × c-MET	4	BLD1102, a novel linker/payload combination; payload is BCPT02, a topoisomerase I inhibitor	I	NCT06515990	80% of patients experienced TRAEs; however, most were grade 1–2; grade ≥ 3 TRAEs reported in 22.2% of patients, including lymphopenia, neutropenia, anemia, and leukopenia.	Likely IgG1 (details not disclosed)	Doma Biopharmaceutical/Biocytogen	First-in-human dose escalation study reported early clinical data on NSCLC and SCLC only, though trial is currently enrolling CRC patients.
JSKN027	PD-L1 × VEGFR2	4	Undisclosed topoisomerase I inhibitor conjugated via cleavable linker	I	NCT07391644	Clinical safety data are not yet available.	IgG1-like (does not disclose silencing mutations)	Jiangsu Alphamab	First-in-human study for patients with advanced solid tumors who have progressed on standard therapy.
JSKN003	HER2 biparatopic	4	Topoisomerase I inhibitor conjugated via DBCO tetrapeptide linker	III I	NCT07384377 NCT05494918	Most TRAEs were grade 1–2; grade ≥ 3 TRAEs were infrequent, with toxicities consistent with other MMAE-containing ADCs and no new safety signals reported.	IgG1	Jiangsu Alphamab	No dedicated CRC trial data yet, but Phase III nearing enrollment for advanced CRC patients who failed to respond to chemotherapy.
Zanidatamab zovodotin (ZW49)	HER2 biparatopic	≈2	ZD02044 (auristatin-based microtubule inhibitor) conjugated via a valine-citrulline cleavable linker	I	NCT03821233	Most adverse events were grade 1–2 (including diarrhea, fatigue, nausea, and infusion-related reactions), with relatively few grade ≥ 3 TRAEs; the recommended phase II dose was successfully established.	Engineered IgG1-like	Zymeworks/Jazz Pharmaceuticals	First-in-human dose escalation study in patients with HER2-positive tumors including CRC.

## Data Availability

No new data were created or analyzed in this study, Data Sharing is not applicable to this article.

## Abbreviations

The following abbreviations are used in this manuscript:

ADC	Antibody–drug conjugate
ADCC	Antibody-dependent cellular cytotoxicity
ADCP	Antibody-dependent cellular phagocytosis
AKT	Protein kinase B
APC	Adenomatous polyposis coli
B7-H3	B7 homolog 3
BiTE	Bispecific T cell engager
BRAF	B-Raf proto-oncogene, serine/threonine kinase
bsAb	Bispecific antibody
bsADC	Bispecific antibody–drug conjugate
CAB	Conditionally active biologic
CAR	Chimeric antigen receptor
CD3	Cluster of differentiation 3
CD8	Cluster of differentiation 8
CD44	Cluster of differentiation 44
CD47	Cluster of differentiation 47
CD133	Cluster of differentiation 133/prominin-1
CD137/4-1BB	Cluster of differentiation 137
CD166	Cluster of differentiation 166/activated leukocyte cell adhesion molecule (ALCAM)
CDC	Complement-dependent cytotoxicity
CDH17	Cadherin-17
CEACAM5	Carcinoembryonic antigen-related cell adhesion molecule 5
CH3	Constant heavy chain domain 3
CLDN18.2	Claudin 18.2
c-MET	Mesenchymal–epithelial transition factor (MET receptor)
CPT2	Camptothecin 2
CRC	Colorectal cancer
CSC	Cancer stem cell
CTLA-4	Cytotoxic T-lymphocyte-associated protein 4
DAR	Drug-to-antibody ratio
dMMR	Deficient mismatch repair
DNA	Deoxyribonucleic acid
DXd	Deruxtecan
EGFR	Epidermal growth factor receptor
EpCAM	Epithelial cell adhesion molecule
EphA-2	Ephrin type-A receptor 2
Fc	Fragment crystallizable region
FcγR	Fc gamma receptor
FOLFIRI	Folinic acid, fluorouracil, and irinotecan
FOLFOX	Folinic acid, fluorouracil, and oxaliplatin
GI	Gastrointestinal
GPCR	G protein-coupled receptor
GUCY2C	Guanylate cyclase 2C
HC	Heavy chain
HER2	Human epidermal growth factor receptor 2
HER3	Human epidermal growth factor receptor 3
HLA	Human leukocyte antigen
IgG	Immunoglobin G
IgG1	Immunoglobin G1
ISC	Intestinal stem cell
KRAS	Kirsten rat sarcoma viral oncogene homolog
LAG-3	Lymphocyte activation gene 3
LC	Light chain
LGR5	Leucine-rich repeat-containing G protein-coupled receptor 5
LY6G6D	Lymphocyte Antigen 6 Family Member G6D
mAb	Monoclonal antibody
MAPK	Mitogen-activated protein kinase
mCRC	Metastatic colorectal cancer
MHC	Major histocompatibility complex
MLH1	MutL homolog 1, mismatch repair system component
MMAE	Monomethyl auristatin E
MMAF	Monomethyl auristatin F
MSH2	MutS homolog 2, mismatch repair system component
MSH6	MutS homolog 6, mismatch repair system component
MSI	Microsatellite instability
MSI-high	Microsatellite instability-high
MSS	Microsatellite stable
MTD	Maximum tolerated dose
NK	Natural killer
NSCLC	Non-small cell lung cancer
PBD	Pyrrolobenzodiazepine
PD-1	Programmed cell death protein 1
PD-L1	Programmed death-ligand 1
PI3K	Phosphoinositide 3-kinase
PI3KCA	Phosphatidylinositol 4,5-bisphosphate 3-kinase catalytic subunit alpha
PMS2	PMS1 homolog 2, mismatch repair system component
RAS	Rat sarcoma (RAS family of small GTPases)
RSL3	RAS-selective lethal 3
RTK	Receptor tyrosine kinase
scFv	Single-chain variable fragment
SCLC	Small cell lung cancer
SMAD4	SMAD family member 4
TCR	T cell receptor
T-DXd	Trastuzumab deruxtecan
TNFR2	Tumor necrosis factor receptor 2
Top1i	Topoisomerase I inhibitor
TP53	Tumor protein p53
TRAE	Treatment-related adverse event
VEGF	Vascular endothelial growth factor
VEGFR2	Vascular endothelial growth factor receptor 2
